# Psychological Aspects of Syncopes and Possible Association with Recurrency—The Role of Implantable Loop Recorders

**DOI:** 10.3390/jpm12081219

**Published:** 2022-07-26

**Authors:** Kevin Willy, Christian Ellermann, Sarah Syring, Benjamin Rath, Florian Reinke, Daniela Willy, Julian Wolfes, Felix K. Wegner, Lars Eckardt, Julia Köbe, Nexhmedin Morina

**Affiliations:** 1 Department for Cardiology II: Electrophysiology, University Hospital Münster, Albert-Schweitzer-Campus 1 Gebäude A1, 48149 Münster, Germany; christian.ellermann@ukmuenster.de (C.E.); s_syri01@uni-muenster.de (S.S.); benjamin.rath@ukmuenster.de (B.R.); florian.reinke@ukmuenster.de (F.R.); julian.wolfes@ukmuenster.de (J.W.); felix.wegner@ukmuenster.de (F.K.W.); lars.eckardt@ukmuenster.de (L.E.); julia.koebe@ukmuenster.de (J.K.); 2 Department of Psychology, University of Münster, 48419 Münster, Germany; morina@uni-muenster.de; 3 Department of Obstetrics and Gynecology, University Hospital Münster, 48419 Münster, Germany; daniela.willy@ukmuenster.de

**Keywords:** syncope, arrhythmia, monitoring, ILR, loop recorder, mental health

## Abstract

Syncopes are a very common phenomenon and have a high recurrence rate. The differentiation between the psychogenic and physical, especially of arrhythmic origin, remains difficult. In many cases, an implantable loop recorder is used for the detection of possible arrhythmias, leading to syncopes. Yet, the existing literature suggests that psychological factors may play a significant role in recurrent syncopes. We aimed at analyzing the potential role of several psychological factors on the recurrence of arrhythmic or non-arrhythmic syncopes. Methods and results: A total of 119 patients, who had received an implantable loop recorder for recurrent syncopes at our center between 01/2018 and 12/2021, participated in this retrospective cohort study. Anxiety, depression and quality of life were assessed using extensively validated questionnaires (GAD-7, PHQ-9 and SF-12). The mean follow-up after loop recorder implantation was 710 ± 430 days and 50% of patients were female. The mean patient age was 54.8 ± 18.6 years. Most patients had no evidence of structural heart disease (84%), and normal LV function (92%). A statistical analysis revealed that the presence of structural heart disease was the strongest predictor for arrhythmic syncope during follow-up. In patients with non-arrhythmic syncopes, we found significantly higher levels of anxiety (GAD-7 score: 2.5 ± 2.6 vs. 4.8 ± 4.3) and depression (PHQ-9 score: 3.9 ± 3.6 vs. 6.8 ± 5.1), and a lower quality of life (SF-12 score: 33.7 ± 6.4 vs. 29.6 ± 7.8). Discussion: We identified factors as contributors to a better identification of patients at risk for arrhythmic as well as non-arrhythmic syncopes. Especially anxious or depressive symptoms may hinted at non-arrhythmic causes of syncope. However, the study was limited by its retrospective design and low patient number. Further trials should likewise combine the diagnostic yield of loop recorders with psychometric evaluations before implantation and combine it with additional diagnostic measures, such as video monitoring, to further examine the role of psychological factors in the pathomechanism and treatment of syncope.

## 1. Introduction

Syncopes are defined as a transient complete loss of consciousness (TLOC) caused by temporary cerebral hypoperfusion, with a rapid onset and short duration, and a spontaneous full recovery [1]. This condition represents a very common phenomenon with a lifetime prevalence of 30–50% [1]. The incidence of recurrent syncopes is high, with approximately 20% in 24 months [2]. In the study by Zimmermann et al., the highest predictive value for the recurrence of syncope was a history of more than three syncopes. More importantly, according to this dataset, patients with three or more syncopes had an unfavorable outcome (i.e., death or major adverse cardiovascular events (MACEs)) [2]. Barbic et al. showed a risk of a syncope relapse after an initial syncope, leading to presentation in an emergency department of over 20% in 5 years [3]. Solbiati et al. reported in a review including 25 studies that the risk for a short-term (10 days) incidence for major cardiac events and death was approximately 10% [4].

The diagnostic approach to elucidate the origin of syncope includes the implantation of implantable loop recorders (ILRs) to be able to correlate paroxysmal ECG abnormalities detected with the loop recorders with clinical symptomatology. For this reason, ILRs have proven their superiority over conventional diagnostic tools such as external loop recorders and Holter ECGs [5,6,7,8]. Solbiati et al. performed a systematic review including 49 studies examining the diagnostic yield of ILRs [9]. The authors underlined that an underlying cause could be found in approximately 50% of patients with unexplained syncopes. In approximately half of these patients, arrhythmia could be identified as the reason underlying the high diagnostic value of these devices. These results have led to a class IA indication for ILR implantation in most patients with unexplained syncopes. In the 2018 ESC Guidelines on diagnosis and management of syncope, if no high-risk circumstances exist (severe structural heart diseases or sudden onset with palpitation immediately followed by syncope), recommend the implantation of a pacemaker or ICD instead [1]. This is further important as subjective heart racing often occurs in the temporal context of syncope and to that effect, harmless and dangerous reasons for these symptoms have to be distinguished. Sinus tachycardia is the most important reason for subjective heart racing, and psychological factors may play a significant role in the offspring, symptom maintenance and treatment efficacy. This knowledge has led to the development of psychocardiology as its own sub-specialty with its own specialized therapies [10]. More importantly, heart racing can also hint at a ventricular arrhythmia origin, which puts patients at serious risk.

In clinical routine, patients with unclear syncopes are often young and present with anxiety and/or depressive symptoms. Linzer et al. reported that syncope itself causes psychosocial impairment comparable to chronic active diseases, such as severe rheumatoid arthritis, independent from comorbid diseases or age [11]. Kouakam reported a very high prevalence of up to 50% for underlying psychiatric comorbidities in patients referred for repetitive unexplained syncopes, especially for anxiety disorders [12]. The authors further reported a 1.7% higher risk for recurrent syncopes during a three-year follow-up of patients with a respective psychiatric disorder. Similar results were reported by Ventura et al., who found even higher rates of psychiatric disorders in 81% of participants of a small cohort of patients presenting with recurrent syncopes [13]. Patients were offered psychotherapeutic treatment after a diagnostic set-up, which was ultimately refused by most patients. Interestingly, patients who received treatment were free of recurrent syncopes during the follow-up, while patients who refused treatment experienced further syncopes, indicating the need for psychological interventions in this respect. A similar observation was reported by Magnavita et al., who showed that the risk for syncope was higher in patients with occupational distress, a low sleep quality and poor mental health. The authors reasoned that health promotion interventions could reduce syncopes via reducing sleep quality and reducing (work-associated) stressors [14].

Yet, our clinical observation is that syncopes often stop after ILR implantation or their frequency of occurrence is reduced. This suggests that the recurrence of syncope may be influenced by psychological factors, such as anxiety, depression, personality traits, quality of life, social comparison or trust in medical personnel or devices. Against this background, we aimed at evaluating psychological factors in patients with implantable loop recorders to examine the extent to which they predict syncopes of cardiac or non-cardiac origin.

## 2. Methods

To investigate the association between psychological factors and recurrent syncopes in patients with implantable loop recorders, we included patients being implanted between January 2018 and November 2021 at our tertiary care center after extensive conservative diagnostic measures, as well as an invasive electrophysiological study, not revealing a cardiac cause of syncope in a retrospective manner. In patients with a typical anamnesis for reflex syncope or orthostatic hypotension, we performed a head-up tilt test. A carotid sinus massage was not regularly performed. Indication for implantation was a single high-risk syncope or multiple unexplained syncopes not cleared up by the diagnostic set-up mentioned before. All ILRs were implanted with a class I or IIa indication according to the current guidelines. Further inclusion criteria were an age over 18 years, availability and willingness for a telephone interview to be performed, good knowledge and understanding of the German language and a complete dataset. Exclusion criteria were insufficient language skills, a mental disability (impossibility of informed consent) and a prior explantation of the ILR without arrhythmia detection leading to ICD or pacemaker implantation. Following approval by the local ethics committee (reference number: 2021-087-f-S), data on patient characteristics and underlying medical history were extracted from patient records. After that, a standardized telephone interview was performed with patients who had given informed consent. The battery consisted of the following validated psychometric tools:

### 2.1. Patient Health Questionnaire (PHQ-9)

The PHQ-9 is a module for the diagnosis of depressive disorders from the Patient Health Questionnaire (PHQ-D), and uses 9 items to assess the presence and severity of depressive symptoms over the last 2 weeks on a 4-point Likert scale [15,16].

### 2.2. Generalized Anxiety Disorder Scale (GAD-7)

The GAD-7 is a brief assessment tool used to evaluate anxiety, and has been used in clinical as well as general populations extensively [17,18]. The questionnaire assesses feelings of anxiety and how often they occurred over the last 2 weeks on a 4-point Likert scale.

### 2.3. Trust in Medical Professionals

The scale used in this trial was a 5-item version, which measures the trust patients put in their physicians [19]. Answers were given on a 5-point Likert scale.

### 2.4. Modified Florida Patient Acceptance Scale (FPAS)

The FPAS was initially developed for patients with implantable cardioverter-defibrillators, and has been used to assess the acceptance of the implanted device and the extent to which it influences daily life. As the items do not focus on specific functions of ICDs, we used a modified version, replacing ICD with the more general term “device”, meaning the ILR. The FPAS initially used 18 items, of which 3 were eliminated in the course of use as the discriminatory power between different psychological constructs was not high enough. Therefore, the version used in our trial comprised 15 items reflecting 4 factors (return to life, device-related distress, positive appraisal and body image concerns), which were answered on a 5-point Likert scale.

### 2.5. Short Version of the Iowa–Netherlands Comparison Orientation Measure (INCOM)

To assess the tendency by patients to compare themselves with others, we used the validated short form of the INCOM, which contains 6 items [20]. The scale differentiates between two comparisons of abilities and comparisons of opinions.

### 2.6. Big Five Inventory (BIF-10)

The BIF-10 allows for the measuring of personality traits with only 10 items. As can be concluded from its name, it was theoretically founded on the five-factor model of personality, also known as the Big Five or OCEAN model (O—openness; C—conscientiousness; E—extraversion; A—agreeableness; N—neuroticism), and each factor was assessed with two items [21].

### 2.7. Short-Form Health Survey (SF-12)

The SF-12 is a non-disease-specific questionnaire using 12 items to assess up to 8 dimensions of health and quality of life (physical functioning, social functioning, role limitation (physical), role limitation (emotional), bodily pain, general health, vitality and mental health) and was developed as a short form of the SF-36, extracting the most specific item(s) for the respective dimension [22].

### 2.8. Statistical Analysis

Categorical variables were expressed as frequencies with percentages, while continuous variables were expressed as means with standard deviations (SDs). Chi-square-test or Fisher’s exact test were used to compare categorical variables and Mann–Whitney U test for continuous variables between patients from different groups. Therefore, we divided the patients into three groups: patients with recurrent syncopes of then-proven arrhythmic cause (group 1), patients with recurrent syncopes without arrhythmic correlation (group 2) or patients without recurrent syncopes (group 3). Multi-variable logistic regression analysis was used to examine which psychological or demographic characteristics were independently associated with recurrent syncopes without pathological ECG findings during follow-up.

### 2.9. Follow-Up

The follow-up duration was approximately 2 years (710 ± 430 days). Patients were scheduled for telemetric monitoring of the ILR every three months, and in case of the occurrence of an arrhythmia transmitted by the ILR or a symptom activation of the patient, at our clinic. In-person aftercare was performed every year, including anamnesis, clinical examination, 12-lead ECG and ILR interrogation.

## 3. Results

**Figure 1:**
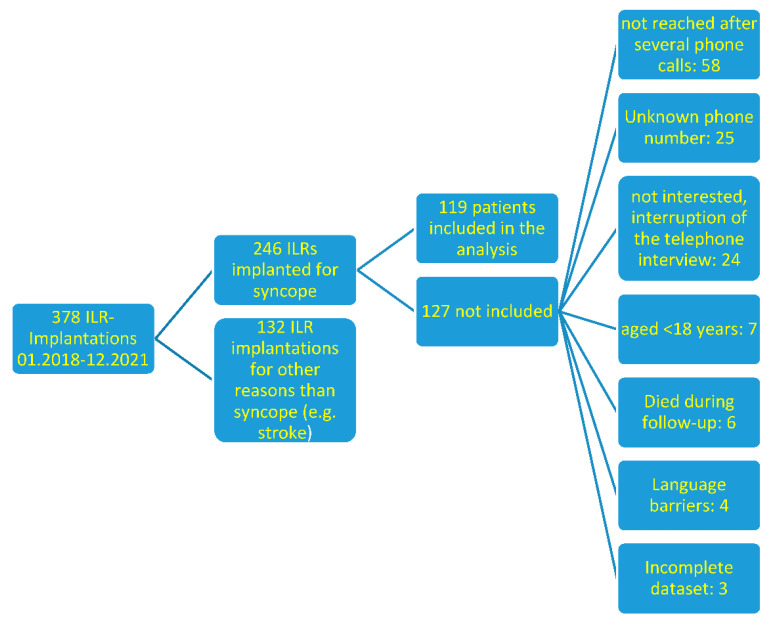
Flowchart of patient evaluation and selection process.

After screening our clinical device implantation registry for implanted loop recorders between January 2018 and December 2021, we identified 378 patients that were possibly suitable for study inclusion. In total, 119 patients were reached out to for a telephone interview and agreed to participate in the study. For detailed information about the patient selection process, please see Figure 1.

The follow-up duration was approximately 2 years (710 ± 430 days), and the gender ratio was approximately 50/50%. The mean patient age was 54.8 ± 18.6 years. Most patients had no evidence of structural heart disease (84%), and normal LV function (92%). A detailed description of patient characteristics is shown in Table 1.

Levels of depressive (5.7 ± 4.5 of a maximum of 36 points) and anxious (4.3 ± 4.1 of a maximum of 28 points) symptoms were recorded with the questionnaires mentioned above. Trust in the medical professionals (18.8 ± 3.8 of possible 25 points) and in the ILR as a device was very high (68.0 ± 7.1 of possible 75 points). There were no relevant body image concerns induced by the ILR (9.9/10 points, lowest score 7/10) and most patients appreciated the ILR as a potential contribution to achieving or restoring a better health status (17.2 ± 2.7 of possible 20 points).

To examine our hypothesis that psychological factors as well as patient characteristics had a predictive value on the recurrence of arrhythmic or psychogenic syncope, we performed linear regression analyses using the recurrence of each syncope type as the dependent variable. There were only two significant factors associated with arrhythmic syncope during follow-up using the variables analyzed. One was the presence of a known structural heart disease at the time of implantation (*p* = 0.04), and the other one was a low level of neuroticism in the BF-10 questionnaire (*p* = 0.02). An association with a low perception of physical functioning reached borderline significance (*p* = 0.07). Concerning the occurrence of non-arrhythmic syncopes, the only significantly associated variable was physical functioning (*p* = 0.02). Two variables reached borderline significance—a low physical role function (*p* = 0.06) and a high value for cooperation (*p* = 0.09). The social comparison orientation and present quality of life did not significantly predict the recurrence of syncope.

In a comparison of three patient groups (patients with recurrent syncopes proven to be arrhythmic (group one), patients with recurrent syncopes without arrhythmic cause (group two) and patients without recurrence (group three)), we showed that levels for anxiety and depressive symptoms were significantly higher in group one than in group two. In addition to that, levels of neuroticism were also higher and quality of life was lower in group two compared to patients from group one. For a more detailed presentation of all results of this analysis, please look at Table 2, Table 3 and Table 4.

## 4. Discussion

In our study, we found a significantly higher level of anxious and depressive symptoms in patients with recurrent syncopes of non-arrhythmic origin and a worse quality of life. However, we did not find single factors serving as possible future predictors of these non-arrhythmic syncopes during the regression analysis, except for the presence of structural heart disease before implantation. This factor was associated with a later arrhythmia in the ILRs, leading to the implantation of either a pacemaker or an ICD, which is in line with existing data [2]. There were no other factors with significant association.

Concerning non-arrhythmic syncopes, a low subjective perception of physical functioning and physical role functioning showed a significant association with the occurrence of non-arrhythmic syncopes during follow-up. This is relevant, as patients suffering from an arrhythmic syncope were exposed to higher levels of structural heart disease and hypertension and, therefore, probably had higher levels of physical impairment than the aforementioned group. Furthermore, patients with non-arrhythmic syncopes had higher levels of anxiety or depressive symptoms than patients with arrhythmic syncopes. Furthermore, non-arrhythmic recurrent syncope was associated with a worse quality of life.

In addition to that, we needed to consider that other psychological factors that we did not examine in our study may play a significant role in the recurrence and risk for syncope. Ferguson showed in different cohorts that fainting and vasovagal syncope had a negative association to intellect, but a positive correlation to perceived threat and the fear of loss. Emotional stability was negatively correlated with the risk of fainting [23]. In our cohort, personality traits, apart from neuroticism, were not associated with the recurrence of syncope or the type of underlying origin, which may also have been due to the small number of patients included. These syncopes might be possibly addressed by psychological interventions, so that a thorough identification of possible stressors and risk factors is mandatory in the assessment of syncopes, in line with the results by Magnavita et al. described in the introduction [14].

In a retrospective analysis, Walsh et al. showed in more than 1400 patients referred to a syncope unit that a history of psychiatric disorders was associated with a later diagnosis of psychogenic pseudosyncope (PPS) [24]. Similar results were reported in the Irish Longitudinal Study of Aging (TILDA) [25], where the authors identified the fear of falling as a major contributor to quality of life and a significant predictor of syncopes, once again underlining the possible potential of psychotherapeutic interventions in the treatment of patients with recurrent syncope [25]. A small pilot trial from Brazil revealed an improvement in quality of life and a reduction in syncope recurrence in a randomized design, further emphasizing the meaningfulness of the approach [26]. In our trial, we assessed many different psychological factors and underlying psychometric concepts for the possible prediction of psychogenic factors leading to syncopes, and could identify an association in non-arrhythmic syncopes to psychological findings such as higher anxiety and depression. These findings might help to improve risk stratification concerning the probability of arrhythmic or non-arrhythmic syncopes. The identification of psychogenic syncopes remains of high importance to provide early diagnosis and organize qualified therapeutic support for these patients. It is, however, very important to underline one of the main results of this study, that a psychogenic syncope still remains a diagnosis by exclusion and that all patients, regardless of underlying psychological pathologies, may not be pre-judged, but have to be carefully examined for underlying causes such as arrhythmias. This is illustrated by the fact that no less than one-fifth of the patients with an ILR were in need of a pacemaker or ICD during a follow-up of approximately two years. The use of the ILR was definitely a strength of this study, as it guaranteed a correct differentiation between different reasons for syncope.

## 5. Limitations

On the other hand, the design was retrospective. The patients were not consecutively included, but depended on their willingness to perform a telephone interview. Furthermore, psychological factors could, for the sake of the study design, only be associated with the later occurrence of arrhythmic or non-arrhythmic syncope, but not serve as true predictors. In addition to that, the trial used a cohort design without a control group to be compared to, which also has to be taken into account when looking at the results.

For an early identification of PPS, a detailed history, optimally supported by an observer, is helpful [27]. However, in our study, we did not use more elaborated diagnostic measures such as video recording during the tilt test, in order to further support the finding of PPS, so that we could discriminate only between syncopes of arrhythmic or non-arrhythmic origin and no other entities within the non-arrhythmic ones.

In addition to that, the number of patients could be too low, leading to statistical power issues and also the possibly of false negative results.

## 6. Conclusions

Future studies should apply longitudinal and prospective designs, maybe also with placebo-controlled designs (reveal implantation vs. sham procedure), to examine potential predictors of non-arrhythmic syncopes and potential facilitators of risk stratification concerning the origin of syncope and the role of ILRs on the psychologic well-being. Equally important are studies examining the role of psychotherapeutic interventions, which may help patients with syncopes with elevated levels of psychopathology. Therefore, the identification of the cause of syncope remains an important challenge that is facilitated by an accurate multi-disciplinary clinical approach and by the correct use of new diagnostic tools, including ILR, as suggested by the recent guidelines. However, the possible underlying psychological mechanisms should also be evaluated during the diagnostic work-up of syncopes and addressed, if necessary, in an equally structured manner.

## Figures and Tables

**Table 1:** Baseline characteristics of the patient cohort included.

Baseline Characteristics	Total (*n* = 119)
Male (n/%)	60 (50.4%)
Age (years)	54.8 ± 18.6
Body height (cm)	176 ± 9
Body weight (kg)	79.6 ± 16.9
BMI (kg/m^2^)	25.7 ± 4.8
Impaired LV-EF (n/%)	10 (8.4%)
Structural heart disease (n/%)	19 (16%)
Arterial hypertension (n/%)	51 (42.9%)
Intake of betablockers (n/%)	40 (33.6%)
Intake of psychotropic drugs (n/%)	14 (11.8%)
Previous psychologic/psychiatric treatment (n/%)	18 (15.1%)
Previous subcutaneous ICD	2 (5%)

**Table 2:** Differences between three patient groups as indicated.

	Group 1: Proven Arrhythmic Cause (*n* = 23)	Group 2: Recurrent Non-Arrhythmic Syncope (*n* = 32)	Group 3: No Syncopes during Follow-Up (*n* = 64)	
Structural heart disease	7 (30.4%)	1 (3.1%)	11 (17.2%)	*p* (1 vs. 2) = 0.01 * p (1 vs. 3) = 0.18 *p* (2 vs. 3) = 0.05 *
Hypertension	8 (34.8%)	15 (46.9%)	28 (43.8%)	*p* (1 vs. 2) = 0.38 *p* (1 vs. 3) = 0.46 *p* (2 vs. 3) = 0.77
Betablocker medication	7 (30.4%)	10 (31.3%)	23 (35.9%)	*p* (1 vs. 2) = 0.95 *p* (1 vs. 3) = 0.46 *p* (2 vs. 3) = 0.65
Prior intake of psychoactive drugs	1 (4.3%)	6 (18.8%)	7 (10.9%)	*p* (1 vs. 2) = 0.88 *p* (1 vs. 3) = 0.35 *p* (2 vs. 3) = 0.30
Prior psychiatric treatment/ psychotherapy	3 (13.0%)	9 (28.1%)	6 (9.4%)	*p* (1 vs. 2) = 0.19 *p* (1 vs. 3) = 0.63 *p* (2 vs. 3) = 0.02 *
Follow-up duration	749 ± 476	760 ± 443	672 ± 409	*p* (1 vs. 2) = 0.93 *p* (1 vs. 3) = 0.46 *p* (2 vs. 3) = 0.34
PHQ-9	3.9 ± 3.6	6.8 ± 5.1	5.8 ± 4.2	*p* (1 vs. 2) = 0.02 * p (1 vs. 3) = 0.06 *p* (2 vs. 3) = 0.27
GAD-7	2.5 ± 2.6	4.8 ± 4.3	4.8 ± 4.4	*p* (1 vs. 2) = 0.03 * p (1 vs. 3) = 0.02 * p (2 vs. 3) = 1.00
Trust in medical professionals	20.0 ± 4.3	18.7 ± 3.5	18.4 ± 3.8	*p* (1 vs. 2) = 0.23 *p* (1 vs. 3) = 0.10 *p* (2 vs. 3) = 1.00
FPAS	70.0 ± 5.6	66.3 ± 8.6	68.0 ± 6.6	*p* (1 vs. 2) = 0.08 *p* (1 vs. 3) = 0.20 *p* (2 vs. 3) = 0.29
Social comparison scale	11.7 ± 5.1	14.2 ± 5.8	12.6 ± 5.3	*p* (1 vs. 2) = 0.11 *p* (1 vs. 3) = 0.50 *p* (2 vs. 3) = 0.18
SF-12 total score	33.7 ± 6.4	29.6 ± 7.8	31.8 ± 6.3	*p* (1 vs. 2) = 0.04 * p (1 vs. 3) = 0.21 *p* (2 vs. 3) = 0.15
BF—extraversion	7.6 ± 2.1	7.1 ± 2.4	7.3 ± 2.3	*p* (1 vs. 2) = 0.41 *p* (1 vs. 3) = 0.56 *p* (2 vs. 3) = 0.71
BF—agreeableness	7.0 ± 2.2	7.1 ± 2.4	6.5 ± 2.1	*p* (1 vs. 2) = 0.92 *p* (1 vs. 3) = 0.32 *p* (2 vs. 3) = 0.23
BF—conscientiousness	8.5 ± 1.7	8.4 ± 2.0	8.5 ± 2.0	*p* (1 vs. 2) = 0.94 *p* (1 vs. 3) = 0.96 *p* (2 vs. 3) = 0.97
BF—neuroticism	3.8 ± 1.8	6.1 ± 2.7	5.3 ± 2.4	*p* (1 vs. 2) = 0.01 * p (1 vs. 3) = 0.01 * p (2 vs. 3) = 0.11
BF—openness	7.8 ± 2.3	6.9 ± 2.5	7.4 ± 2.6	*p* (1 vs. 2) = 0.17 *p* (1 vs. 3) = 0.48 *p* (2 vs. 3) = 0.38

Categorial variables are presented as frequencies and percentages of the whole population. Means and standard deviations (SDs) are expressed for the respective psychometric questionnaire. *p*-values for multiple comparisons between groups are shown on the right-hand side. Significant *p*-values were defined as *p* < 0.05 and were indicated with an asterixis (*).

**Table 3:** Arrhythmic causes for recurrent syncopes detected by the ILR.

Type of Arrhythmia	*n* = 23
Symptomatic VT and structural heart disease	4 (17.4%)
Symptomatic high-degree AV block (IIb° or III°)	7 (30.4%)
Symptomatic sinus arrest	12 (52.2%)

Display of the arrhythmic events leading to ICD or pacemaker implantation in patients from group one expressed as means with percentages.

**Table 4:** Results of the multivariable logistic regression analysis.

Parameter	*p*-Value (Occurrence of Arrhythmic Syncopes)	*p*-Value (Occurrence of Non-Arrhythmic Syncopes)
Age	0.271	0.181
Sex	0.172	0.663
Structural heart disease	0.043 *	0.105
Impaired LV function	0.522	0.961
Arterial hypertension	0.100	0.187
Betablocker intake	0.295	0.728
Psychotropic agent intake	0.396	0.173
Prior psychological treatment	0.185	0.868
Number prior syncope	0.405	0.800
PHQ-9 score	0.943	0.650
GAD-7 score	0.542	0.635
SF-12 score	0.795	0.460
BF-10 neuroticism	0.017 *	0.155
BF-10 extraversion	0.501	0.955
BF-10 conscientiousness	0.488	0.481
BF-10 openness	0.510	0.525
BF-10 agreeableness	0.645	0.088

Results of the regression analysis for the examined variables with arrhythmic as well as non-arrhythmic syncopes with respective *p*-values. Significant *p*-values were defined as *p* < 0.05 and were indicated with an asterixis (*).

## Data Availability

Additional and raw data can be obtained from the corresponding author under kevin.willy@ukmuenster.de upon reasonable request.
